# G.A study protocol for a randomized controlled trial investigating the influence of Iyengar Yoga on biofunctional age and cardiovascular risk associated biomarker of postmenopausal women

**DOI:** 10.3389/fgwh.2026.1762048

**Published:** 2026-03-16

**Authors:** Simona Ott, Elena Pavicic, Sofie Schelker, Rebecca Re, Kerstin Khattab, Petra Stute

**Affiliations:** 1 University of Bern, Bern, Switzerland; 2 Gynecologic Endocrinology and Reproductive Medicine, University Clinic of Obstetrics and Gynecology, Bern, Switzerland; 3 Graduate School for Health Sciences, University Hospital of Bern, Bern, Switzerland; 4 HerzRaum, Integrative Centre for Yoga and Heart Health, Zürich, Switzerland

**Keywords:** aging, cardiovascular diseases, c-Reactive protein, menopause, oxidativestress, postmenopause, yoga

## Abstract

**Background:**

Previous research has shown that women aged 45–54 experience a significantly faster rate of biological aging compared to the preceding or following decade. Exactly in this period of life women will experience the menopause. Menopause is defined as the last menstrual period in a women's life and is diagnosed retrospectively after 12 month of amenorrhea. The menopausal transition is associated with a reduced overall well-being. Practicing yoga has demonstrated beneficial effects on cardiovascular risk factors such as BMI, lipid profile, HbA1c, thus contributing to better overall well-being. Therefore, yoga may represent a promising intervention to support healthy aging in postmenopausal women and may positively influence cardiovascular-related biomarkers.

**Objective:**

The primary objective of this study is to assess the effect of Iyengar Yoga on the biofunctional age (BFA) of postmenopausal women, assessed using the biofunctional status (BFS). The secondary objectives are to evaluate its impact on the frequency of hot flushes, high-sensitivity C-reactive protein (hsCRP) levels, and oxidative stress.

**Methods:**

This paper describes the study protocol of a monocentric, randomized, controlled, open-labeled, two-arm, interventional study conducted at the University Hospital of Bern, Switzerland. A total of 72 postmenopausal women will be randomly assigned to either an intervention group or a control group. Participants in the intervention group will attend a 90-minute Iyengar yoga class once per week for 12 weeks, guided by a certified instructor. Additionally, they will perform two 45-minute home-based yoga sessions per week, including postures and relaxation techniques. The control group will not receive any intervention during this period.

**Conclusion:**

This protocol describes a randomized controlled trial evaluating whether Iyengar Yoga can reduce the biofunctional age (BFA), calculated using the BFS, of postmenopausal women aged 45–54 years in 12 weeks. Furthermore, yoga practice may have a positive impact on cardiovascular risk factors and overall vitality.

**Clinical Trial registration:**

The study has been registered at Clinicaltrials.gov (Identifier: NCT04705623) on December 30, 2020. https://clinicaltrials.gov/study/NCT04705623

## 1 Introduction

Aging is an inevitable biological process that all humans experience (1). With increasing age, women undergo the menopausal transition, which typically occurs between the ages of 45 and 52 (2, 3). It is driven by hormonal changes and may negatively affect a woman's overall well-being (2). Approximately 75% of the women report climacteric symptoms such as hot flushes, night sweats, sleep disturbances, and mood changes including depressive symptoms (2, 4). Menopause is defined as the final menstrual period in a woman's life and can only be confirmed retrospectively, after 12 consecutive months of amenorrhea without any other pathological or physiological cause (5).

During the decade of life in which women typically experience menopause, aging has been shown to accelerate, as measured by the biofunctional status (BFS) (6). The BFS reflects an individual's overall functional capacity and serves three purposes: assessing health risks and resources, guiding health-promoting interventions, and evaluating aspects of age, health, and disease (7). Based on the BFS, the biofunctional age (BFA) can be calculated, providing a measure of functional aging and resources for healthy aging (8).

According to the World Health Organization, healthy aging can be promoted through regular physical activity and a balanced diet (1). Yoga represents one possible form of physical activity. Iyengar Yoga, the style used in this study, is the most widely practiced form of yoga in Western countries (9) and seeks to achieve a state of equilibrium by cultivating endurance, relaxation, strength, and stability throughout the entire body (9). Several studies and meta-analyses report beneficial effects of yoga on cardiovascular risk factors (e.g., BMI, lipid profiles, HbA1c) (10), physical function (11, 12), as well as stress reduction (13).

Cardiovascular risk associated biomarker and inflammatory markers such as high-sensitivity C-reactive protein (hsCRP) can serve as predictors of arterial hypertension and cardiovascular disease (12, 14). In premenopausal women, hs-CRP levels are lower than in postmenopausal women (12). Higher hs-CRP levels are associated with a greater prevalence of arterial hypertension among all midlife women (12).

Given these numerous positive effects of yoga on various symptoms, we hypothesize that yoga will also have a beneficial effect in postmenopausal women.

The primary objective of this trial is to investigate the positive change in BFA of postmenopausal women participating in a 12-week Iyengar Yoga intervention compared with a control group that does not receive yoga classes.

The secondary objectives include the analysis of changes in climacteric symptoms, with a particular focus on hot flushes. We expect that the intervention will reduce the frequency of hot flashes as well as decreased hs-CRP levels and oxidative stress in the intervention group.

## 2 Methods

### 2.1 Trial design

This clinical trial is a monocentric, randomized, controlled, open-labeled, two-arm, interventional study.

### 2.2 Trial setting

The study is conducted at the Department of Gynecology, University Hospital of Bern. This study employs a hybrid design, with data collection conducted at the University Hospital of Bern and the intervention delivered both at participants’ homes and at a yoga studio in Bern. The Cantonal Ethics Committee of Bern (KEK: 2019-01794) approved the study on March 27, 2023 (Supplementary File S1). The objective of this clinical trial is to evaluate the effect of Iyengar Yoga on the BFA of postmenopausal women. The study protocol has been prepared and reported in accordance with the SPIRIT guidelines (15) (Supplementary File S2).

### 2.3 Inclusion and exclusion criteria

Participants were eligible for this study if they provide written informed consent and were postmenopausal. Postmenopause is defined as either 12 months of spontaneous amenorrhea, six months of spontaneous amenorrhea with serum follicle stimulating hormone levels greater than 40 mIU/mL, or six weeks after a bilateral oophorectomy with or without hysterectomy. We included only postmenopausal women because the definition is clear, allowing for a more homogeneous study population. Eligible participants must experience at least four hot flushes per day, as assessed using the Menopause Rating Scale II questionnaire (16). They must not have practiced yoga regularly in the past two years. Participants must be good German speakers and willing to attend all 12 guided yoga classes as well as two 45-minute home-based yoga sessions per week. We included only German-speaking participants, as the BFS was developed in German and this ensures that there are no comprehension issues. In addition, they must agree to attend two BFS assessments and to provide blood samples.

Participants are excluded from the study if they have had an acute or serious disease within the past two years, such as cancer or major surgery, or if they have autoimmune or chronic inflammatory diseases, including rheumatism or thyroid dysfunction. Individuals with mental illnesses, such as depression or anxiety disorder, measured with the Hospital Anxiety and Depression Scale score (17), acute or chronic back pain, or herniated discs are also excluded. Additional exclusion criteria include current hormone replacement therapy, smoking more than 20 cigarettes per day or having a history of more than 20 pack-years, and alcohol consumption exceeding 30 g per day (equivalent to more than 1 L of beer or 0.3 L of wine). Individuals who have participated periodically in yoga classes within the last two years, those unable or contraindicated to undergo the intervention, or participants incapable of making informed judgments or under legal tutelage are also excluded.

### 2.4 Informed consent

All participants receive detailed written information about the study and provide written informed consent before any study procedures begin. A minimum of 24 h is granted for consideration prior to consent (Supplementary File S3).

### 2.5 Intervention group

The study intervention consists of a 90-minute yoga session per week conducted over a period of 12 weeks. Certified advanced junior instructors in Iyengar Yoga will deliver the yoga session online to increase scheduling flexibility and minimize participant absences.

Each session is structured into two parts: the first part focuses on active postures (asanas), followed by relaxation and breathing exercises in the second part. The yoga instructors have designed the program specifically for postmenopausal women experiencing climacteric symptoms, based on the book “Iyengar Yoga in der Menopause” (18).

In addition to the weekly group session, participants are required to perform two home-based yoga sessions per week, each lasting 45 min. These sessions are divided into 30 min of postures and 15 min of relaxation techniques, introduced during the instructor-led class. Participants in the intervention group will also receive a photo sequence of the exercise routine for home practice (Supplementary File S4).

Participants may perform the two home sessions either consecutively or at separate times, according to personal preference. Participants receive the phone number of the yoga instructor to address any questions that may arise during the home-based intervention.

### 2.6 Control group

The control group will receive no intervention. We have chosen a control group to isolate the specific effects of yoga practice. Upon completion of the 12-week intervention or control period, all participants will undergo the second examination.

### 2.7 Adherence

To minimize loss to follow-up, the study staff reminds the participants for assessments and home-based yoga sessions by phone. At the end of the study, each participant will receive the results of her individual BFS assessments, showing changes before and after the 12-week period, along with a personal consultation with Prof. Petra Stute, who will review the BFS results and provide guidance on how to strengthen and vitalize individual health resources. Participants in the control group will be offered a complimentary yoga session after the second assessment. This feedback is intended to enhance adherence and demonstrate the personal value of participation.

### 2.8 Primary and secondary outcomes

The primary endpoint of this trial is the change in BFA of participants enrolled in this randomized controlled study, calculated at baseline and after the 12-week intervention period. To evaluate BFA, the study staff will conduct a BFS assessment for each participant, comprising 45 different noninvasive tests performed at University Clinic of Obstetrics and Gynecology, Inselspital Bern.

The secondary endpoint includes the assessment of changes in climacteric symptoms, particularly hot flushes, and alterations in hsCRP levels and oxidative stress. These parameters will be measured concurrently with the BFS assessments for each participant. Variations in participant adherence and engagement may influence both the primary and secondary end points, for example due to lack of motivation or limited time availability. Furthermore, potential imbalances in the frequency or quality of home-based yoga sessions could affect the significance of the study outcomes.

### 2.9 Measurements

The BFS is an age- and gender-validated, noninvasive assessment of vitality. It comprises 45 standardized tests that evaluate psychological, physical, and social parameters (Supplementary File S5). In addition to the individual results (BFS), an established calculation algorithm can be applied to generate an integrative sum score, the BFA. The calculation algorithms are based on the model developed at the Max Bürger Aging Research Centre (University of Leipzig) (19) for operationalizing vitality and functional age.

Secondary measurements include blood, urine, and saliva samples, which nursing staff will collect at both assessment points (baseline and postintervention). We will analyze the following parameters in blood: LDL cholesterol, HDL cholesterol, triglycerides, total cholesterol, hs-CRP, oxidized LDL, oxidized and reduced glutathione, FSH, and estradiol (E2), as well as 8-isoprostane in urine. These biomarkers will be used to evaluate hs-CRP levels and oxidative stress, thereby assessing the potential physiological effects of the yoga intervention. The participants also complete the Menopause Rating Scale II questionnaire (16) to assess climacteric symptoms.

### 2.10 Participant timeline

The entire project will span two years. Following the screening procedure, participants will attend two in-person visits (see Table 1). The intervention group will participate in a 12-week Iyengar yoga program, while the control group will not receive any intervention. A second assessment will be conducted thereafter.

**Table 1:** SPIRIT schedule of enrolment, interventions and assessment.

Visit	Information	Screening	First visit	Intervention (12 weeks)	Second visit
Visit week	0	1	2	2-14	14
Patient information	+				
Written consent		+			
Medical history		+			
Physical examination		+			
Participant characteristics		+			
Biofunctional status			+		+
Intervention or control				+	
Questionnaire		+	+		+
Sampling			+		+
Safety		+	+		+

### 2.11 Sample size and power calculation

We will include a maximum of 72 postmenopausal women experiencing at least four hot flushes per day. The study staff randomly assign participants to one of this two groups: an intervention group comprising up to 36 participants receiving the Iyengar Yoga classes, and a control group comprising up to 36 postmenopausal women who will not receive any intervention. The sample size of 36 participants per group is sufficient to detect a difference with an effect size of 0.75 at a significance level of 5% (two-tailed) and a statistical power of 80%, as calculated using the nonparametric Mann–Whitney *U*-test.

### 2.12 Enrollment procedure

Postmenopausal women eligible for participation in this study are either patients of University Clinic of Obstetrics and Gynecology, Inselspital Bern, or are recruited through public advertisements (e.g., the Inselspital website, Yoga—Das Magazin, flyers, Yogavereingung Bern, or local gynecologists in Bern).

Patients of the clinic who experience at least four hot flushes per day will be informed by their attending physicians about the opportunity to participate in the clinical trial and will receive a written information sheet.

### 2.13 Allocation

A computer has generated the randomization codes to ensure unbiased group allocation. These codes are printed on separate sheets of paper and placed in sealed, opaque envelopes to maintain allocation concealment.

The document containing the randomization list is saved in Excel format and securely stored on a password-protected computer at University Clinic of Obstetrics and Gynecology, Inselspital Bern.

### 2.14 Blinding

Participants and members of the treatment team are not blinded to group allocation, as blinding was not feasible due to the behavioral nature of the intervention.

### 2.15 Confidentiality and data management

We handle all trial and participant data with the most discretion. Only authorized personnel will have access to the data necessary to perform their duties within the scope of the study. On case report forms and other study-specific documents, participants will be identified solely by a unique participant number.

The server hosting the electronic data capture system and database is kept in a locked server room, with direct access restricted to system administrators. A role-based access system with personal passwords regulates user permissions according to their responsibilities.

All data entered the case report forms are transferred to the database using Secure Sockets Layer encryption. Each data point is tagged with the identity of the user entering it, along with the exact date and time. Any retrospective changes to data in the database are logged in an audit trail, recording the time, table, data field, original and modified values, and the responsible user.

A multi-level backup system is implemented. Internal backups of the entire system, including the database, are performed multiple times per day, with additional daily external backups stored in a secure location in a separate building.

Biological material collected in this study is labeled only with the unique participant number, not the participant's name, and is stored securely in a restricted-access area accessible only to authorized personnel.

### 2.16 Risks

Participants face minimal risk. Blood draws by trained staff may rarely cause infection, hematoma, or nerve injury. Yoga sessions are supervised by certified instructors. The risk of injury is minimal, since the yoga sessions are designed specifically for postmenopausal women. Participants may contact the yoga instructor by phone at any time should any issues arise during the online or self-directed sessions. No posttrial care is planned.

### 2.17 Statistical analysis

For the primary outcome, the BFA, we will use the nonparametric Mann–Whitney *U*-test. The 45 individual BFS tests will be evaluated exploratively as secondary variables.

To maintain consistency between the sample size calculation and the planned analysis, the significance level for the primary analysis will be set at 0.05 (two-sided), which aligns with the assumptions used to estimate the required sample size. Rejection of the null hypothesis would indicate that a weekly 12-week yoga intervention significantly reduces BFA in postmenopausal women. The sample size of 36 participants per group (intervention and control) provides 80% power to detect an effect size of 0.75 using a two-sided Mann–Whitney *U*-test.

The statistician will perform the data analysis using IBM SPSS software and will compare all groups with respect to outcome variables and covariates. The analysis will be performed according to the intention-to-treat principle.

### 2.18 Withdrawal and discontinuation

Participants are withdrawn from the study if they are newly diagnosed with a serious disease or if they miss one or more assessments, or if they withdraw their informed consent

If a participant withdraws from the study or discontinues the trial, we won't replace them.

### 2.19 Missing data and drop-outs

In cases of missing data, we will use the available data to calculate correlation coefficients; missing values will not be replaced. A dropout rate of up to 15% is considered acceptable to maintain statistical significance. Data collected up to dropout will be used for analysis.

### 2.20 Adverse events

Both the Investigator and the Sponsor-Investigator assess the causality of each adverse event with respect to the trial intervention. Events assessed as possibly, probably, or definitely related are classified as related to the intervention, following the International Council for Harmonization E2A guidelines (20).

The Sponsor-Investigator assesses the severity of each adverse event as mild, moderate, or severe. If there is a severe adverse event related to the trial intervention, the Investigator reports the severe adverse event to the Ethics Committee via Business Administration System for Ethics Committees within 15 days.

### 2.21 Amendments

Substantial amendments to the study design, organization, protocol, or relevant study documents must be submitted to the Ethics Committee for approval prior to implementation.

In emergencies, the investigator may deviate from the protocol without prior approval to protect the rights, safety, and well-being of participants. The study staff documents and reports any of these deviations to the Ethics Committee as soon as possible. Substantial amendments affect the safety, health, rights, or obligations of participants, impact the study objectives or the central research question, or involve a change of study site, principal investigator, or sponsor (ClinO, Art. 29) (21).

### 2.22 Monitoring

To ensure accurate, complete, and reliable data, the study site and procedures may be reviewed by independent monitors. Participants’ data privacy is strictly maintained. All study records and source documents are also available to auditors and inspectors, including the Ethics Committee, with queries addressed by the investigator or designated staff.

## 3 Discussion

The present study aims to investigate whether yoga can positively influence the health and well-being of women during the menopausal transition. Previous research has shown that women between 45 and 54 years of age experience an accelerated aging process compared to younger and older age groups (6). By evaluating changes in the BFS before and after a 12-week Iyengar Yoga intervention, this study seeks to determine whether yoga can slow biofunctional aging and thus contribute to healthier aging in postmenopausal women. In addition to biofunctional parameters, the study explores the effects of yoga on climacteric symptoms, particularly hot flushes, which affect women during menopause (5). If the intervention demonstrates a reduction in such symptoms, yoga could be considered a valuable, nonpharmacological strategy to enhance quality of life and vitality in this population.

Previous studies have demonstrated that yoga can positively impact physical, psychological, and cardiovascular health (3, 10, 22). The use of yoga to reduce climacteric symptoms in postmenopausal women aligns with prior research showing benefits of yoga on chronic disease symptoms (3), stress reduction (22), and cardiovascular risk factors (10). This study extends these findings by investigating the impact of yoga specifically on biofunctional aging during the critical menopausal decade.

One limitation of this trial is the relatively small sample size and the short intervention duration, which may affect statistical power and limit the ability to detect small effect sizes. However, the intervention period was intentionally designed to be brief in order to enhance feasibility and participant adherence. To compensate for the shorter duration, the intervention emphasizes a higher practice intensity over the 12-week period. Moreover, the relatively short intervention period may support adherence to the prescribed yoga sessions. Monitoring is also a limitation of the study. To encourage complete and diligent participation, study staff conduct weekly phone calls; however, monitoring adherence remains challenging. An additional limitation concerns the control group, which does not receive an intervention. Participants in the control group are not actively monitored during the 12-week period, which may introduce expectation and attention biases. Furthermore, due to the interactive nature of the yoga intervention, blinding of participants and study personnel was not feasible, potentially further increasing expectation-related bias.

Despite this limitations, both the concept of BFS and the practice of yoga are applicable across a wide range of age groups and health conditions, supporting the broader generalizability of the study findings.

The risk associated with participation is minimal, as the intervention is noninvasive and participants may withdraw at any time without consequence. Providing participants with individual feedback on their BFS results may further support adherence and motivation throughout the study period.

If a positive effect on the BFS is observed, the results could suggest that yoga supports healthy aging in postmenopausal women. Additionally, these findings, together with positive effects on hsCRP and oxidative stress, would contribute to the growing body of evidence indicating that regular yoga practice provides broader benefits for physical, mental, and cardiovascular health (3, 10, 22). If positive effects on climacteric symptoms are also observed, the results could further demonstrate that yoga not only influences functional aging but also alleviates menopause-related complaints.

## Supplementary material

The Supplementary Materialfor this article can be found online at: https://www.frontiersin.org/articles/10.3389/fgwh.2026.1762048/full#supplementary-material

Supplementary File S1 Approval No. 2019-01794 by the Swiss Ethics committee (.pdf)

Supplementary File S2 SPIRIT 2013 Checklist: Recommended items to address in a clinical trial protocol and related documents (.pdf)

Supplementary File S3 Informed Consent form in German (.pdf)

Supplementary File S4 Program of the Yoga-Sessions made at home (.pdf)

Supplementary File S5 Biofunctional Status (.pdf)
