# Supplementary material for: G.A study protocol for a randomized controlled trial investigating the influence of Iyengar Yoga on biofunctional age and cardiovascular risk associated biomarker of postmenopausal women
Source: Front Glob Womens Health. 2026 Mar 16;7:1762048. doi: 10.3389/fgwh.2026.1762048 (PMC13033725; doi:10.3389/fgwh.2026.1762048)
Supplement: Supplementary File S1 — Approval No. 2019-01794 by the Swiss Ethics committee (.pdf) [file Datasheet1.pdf]

Kanton Bern
Canton de Berne

Gesundheits-, Sozial- und Integrationsdirektion
Kantonale Ethikkommission für die Forschung

Murtenstrasse 31
3010 Bern
Bern
+41 31 633 70 70 (Telefon)
+41 31 633 70 71 (Telefax)
info.kek@be.ch
www.be.ch/gsi

Dorothy Pfiffner
+41 31 633 70 77
dorothy.pfiffner@be.ch

GSI-KEK, Murtenstrasse 31, 3010 Bern

Frau
Prof. Dr. med. Petra Stute
Universitätsklinik für Frauenheilkunde
Inselspital Bern
Friedbühlstrasse 19
3010 Bern

Bern, 17. März 2023, MN

Bewilligung Amendment der Kantonalen Ethikkommission Bern

**Wesentliche Änderung**	Amendment inkl. Verlängerung bis 31.12.2023
**eingereicht am**	27.02.2023
**Project-ID**	2019-01794
**Projekttitel**	Influence of Iyengar yoga on the bio-functional age of postmenopausal women
**Master-/Doktorarbeit von**	Schelker, Sofie
**Haupt-Prüfer**	Prof. Dr. med. Petra Stute
**Sponsor**	Prof. Dr. med. Petra Stute
**Zentren**	Prof. Dr. med. Petra Stute, Universitätsklinik für Frauenheilkunde, Inselspital Bern

Entscheidverfahren

☐ vereinfachtes Verfahren ☒ Präsidialverfahren

Entscheid

Prof. Dr. med. Petra Stute, Universitätsklinik für Frauenheilkunde, Inselspital Bern

☒ Die Bewilligung wird erteilt

Bemerkung:

Flyer: Hier ist noch ein Datum aufgeführt: *Der entsprechende Link wird Ihnen Ende Oktober per Email zugesendet*

Gebühren

Betrag CHF 200.– **Tarifcode** 3.3.1

Gemäss der geltenden Gebührenordnung von swissethics. Rechnungsstellung folgt durch die Kantonale Gesundheits-, Sozial- und Integrationsdirektion (GSI).

Rechtsmittelbelehrung

Gegen diese Verfügung kann innert 30 Tagen seit Eröffnung bei der Gesundheits-, Sozial-und Integrationsdirektion des Kantons Bern Beschwerde erhoben werden. Die Beschwerdefrist kann nicht verlängert werden. Die Beschwerdeschrift ist im Doppel bei der Gesundheits-, Sozial-und Integrationsdirektion, Rathausplatz 1, Postfach, 3000 Bern 8 einzureichen.

Eine allfällige Beschwerde, die in mindesten zwei Exemplaren einzureichen ist, muss einen Antrag, die Angabe von Tatsachen und Beweismitteln, eine Begründung sowie eine Unterschrift enthalten; der angefochtene Entscheid und andere greifbare Beweismittel sind beizulegen.

Sie muss

(a) angeben, welche Entscheidung anstelle der angefochtenen Verfügung beantragt wird und
(b) aus welchen Gründen diese andere Entscheidung verlangt wird sowie
(c) die Unterschrift der beschwerdeführenden Partei oder der sie vertretenden Person enthalten.

Der Beschwerdeschrift beizulegen sind die Beweismittel, soweit sie greifbar sind, und die angefochtene Verfügung. (Art. 32 und 60 ff. des Gesetzes vom 23. Mai 1989 über die Verwaltungsrechtspflege [VRPG; BSG 155.21]).

Kopie an

☐ BAG

☒ Andere Sofie Schelker, sofie.schelker@students.unibe.ch

Die Ethikkommission bestätigt, dass sie nach ICH-GCP arbeitet.

Unterschriften

Prof. Dr. med. Christian Seiler<br>Präsident KEK Bern	Dr. sc. nat. Dorothy Pfiffner<br>Vizepräsidentin<br>Leiterin Wissenschaftliches Sekretariat

Anhang: -Bedeutung des Entscheids
-Liste der Dokumente, eingereicht am 27.02.2023

Anhang

Bedeutung des Entscheids

Die Bewilligung wird erteilt: Das Vorhaben kann gemäss der oben aufgeführten wesentlichen Änderung weitergeführt werden.

Liste der Dokumente, eingereicht am 27.02.2023

**Prof. Dr. med. Petra Stute, Universitätsklinik für Frauenheilkunde, Inselspital Bern**		
Dokument	Dok.Datum	Version
**1. Cover Letter**		
cover-letter-antrag-14-02-2023.pdf	14/02/2022	
**3. Participant information sheet and informed consent (ICF)**		
14-02-2023-icf-korrektur.pdf	14/02/2022	8
14-02-2023-icf-korrektur.docx	14/02/2023	8
**4. Study plan (protocol), signed and dated**		
14-02-23-study-protocol-influence-of-iyengar-yoga-on-the-bio-functional-age-of-postmenopausal-women-korrektur-99.pdf	14/02/2023	7
14-02-23-study-protocol-influence-of-iyengar-yoga-on-the-bio-functional-age-of-postmenopausal-women-korrektur-47.docx	14/02/2023	7
**4a. Monitoring plan**		
see doc/cat: **4**, page/ref: **19**		
**5. CRF (Case Report Form)**		
20-07-2022-crf-influence-of-iyengar-yoga-on-the-bio-functional-age-of-postmenopausal-women.pdf	20/07/2022	5
20-07-2022-crf-influence-of-iyengar-yoga-on-the-bio-functional-age-of-postmenopausal-women-korrektur.docx	20/07/2022	5
**6. Investigator's CV, dated**		
cv-prof-dr-med-petra-stute.pdf	20/09/2019	
**7. Investigator's proof of GCP training**		
gcp-prof-dr-med-petra-stute.pdf	20/09/2019	
**10. Insurance**		
see doc/cat: **4**, page/ref: **15**		

11. Other documents handed over to study participants

14-02-2023-flyer-yogastudie.pdf	14/02/2023	6
u-bungssequenz-fu-r-zuhause-im-rahmen-der-menopausenstudie.pdf	14/02/2023	1
14-02-2023-information-fu-r-teilnehmerinnen-des-yogaprogramms-im-rahmen-der-studie-45.pdf	14/02/2023	3
14-02-2023-flyer-yogastudie-korrektur.docx	14/02/2023	6
14-02-2023-information-fu-r-teilnehmerinnen-des-yogaprogramms-im-rahmen-der-studie-45.docx	14/02/2023	3

12. Details on nature and scope/value of compensation for participants

There is no compensation for the participation in this study

14. Information on secure handling of biological material and personal data, and in particular on the storage thereof

see doc/cat: **4**, page/ref: **17-18**

39. Miscellaneous / Varia

24-11-2019-participation-sheet-of-yoga-class-and-check-of-participant-for-yoga-instructor.pdf	24/11/2019	1
masterarbeitsvereinbarung.pdf	20/07/2022	1
24-11-2019-personal-study-plan-and-participants-diary.pdf	24/11/2019	1
