# Supplementary material for: G.A study protocol for a randomized controlled trial investigating the influence of Iyengar Yoga on biofunctional age and cardiovascular risk associated biomarker of postmenopausal women
Source: Front Glob Womens Health. 2026 Mar 16;7:1762048. doi: 10.3389/fgwh.2026.1762048 (PMC13033725; doi:10.3389/fgwh.2026.1762048)
Supplement: Supplementary File S3 — Informed Consent form in German (.pdf) [file Datasheet3.pdf]

Einfluss von Hatha-Yoga auf das biofunktionelle Alter und kardiovaskuläre Risiko von Frauen nach der Menopause

Influence of hatha yoga on the bio-functional age and cardiovascular risk of postmenopausal women

Diese Studie ist organisiert durch die Universität für Frauenheilkunde des Inselspitals, Bern.

Sehr geehrte Interessentin

Gerne möchten wir Sie anfragen, ob Sie an einer klinischen Studie teilnehmen wollen. Im Folgenden wird Ihnen dieses Studienvorhaben dargestellt.

Information

1. Ziel der Studie

Wir möchten in dieser Studie untersuchen ob Iyengar-Yoga einen verjüngenden Effekt auf Frauen nach der Menopause hat. Mit dem biofunktionellen Status, der aus 45 verschiedenen Tests besteht, berechnen wir dazu Ihr biofunktionelles Alter vor Beginn der Studie und nach einem regelmässigen Yogaprogramm über 12 Wochen.
Ausserdem untersuchen wir den Einfluss von Iyengar-Yoga auf Hitzewallungen nach der Menopause und bestimmen den Östrogenspiegel im Blut. Östrogen ist ein Hormon, das zum Teil für die Menopause verantwortlich ist, aber auch den Stoffwechsel beeinflusst. Wir bestimmen durch die Blutentnahme ebenfalls Marker, die als Risikofaktoren für die Entstehung einer Verengung der Herzkranzgefässe gelten. Bei allen diesen Faktoren, die wir messen, interessiert uns ob wir durch das Iyengar-Yoga eine positive Veränderung erzielen können.

2. Auswahl

Es können alle Frauen teilnehmen, die über 12 Monaten ihre Periode nicht mehr gehabt haben und an Hitzewallungen (min. 4/Tag) leiden. Ausserdem müssen sie Deutsch sprechen. Zudem dürfen Sie in den letzten 2 Jahren noch nicht regelmässig Yoga ausgeübt haben und bereit sein an allen Yogastunden teilzunehmen. Nicht teilnehmen hingegen dürfen Personen, die:

- In den letzten 2 Jahren an einer akuten oder schweren Krankheit gelitten haben (z.B. Krebs, grössere Operation)
- An einer autoimmun oder chronisch entzündlichen Krankheit leiden (z.B. Rheuma, Schilddrüsenüber- oder -Unterfunktion)
- An einer psychischen Erkrankung leiden (z.B. Depression oder Angststörung)
- An akuten oder chronischen Rückenschmerzen leiden
- Unter Hormonbehandlung stehen
- Über 20 Zigaretten pro Tag rauchen und/oder regelmässig Alkohol konsumieren (mehr als 1l Bier oder >0.3dl Wein pro Tag).

3. Allgemeine Informationen

Dies ist eine nationale, monozentrische, einfachblinde (Untersucherblinde) und randomisierte Studie. Erklärung zu den Begriffen siehe Punkt 17.

Die Studie wird gemäss den gesetzlichen Bestimmungen der Schweiz durchgeführt. Ausserdem beachten wir alle international anerkannten wissenschaftlichen Richtlinien. Die zuständige Kantonale Ethikkommission hat die Studie geprüft und bewilligt.
Eine Beschreibung dieser Studie finden Sie auch auf der Internetseite des Bundesamtes für Gesundheit: www.kofam.ch

4. Ablauf

4.1 Die Rekrutierung der Teilnehmerinnen für die Studie wird ab August 2022 beginnen. Wenn Sie sich für die Teilnahme bei uns anmelden, kommen Sie zunächst auf eine Warteliste.

4.2 Vor der Studie durchlaufen alle Interessentinnen (unabhängig von der Studienteilnahme) eine Voruntersuchung (Screening), welches an der Frauenklinik im Inselspital durchgeführt wird und ca. eine Stunde dauert. Der Inhalt der Voruntersuchung wird im Kapitel 4.7 genauer beschrieben. Für das Screening erhalten Sie ein Aufgebot und werden dann direkt im Anschluss informiert, ob Sie an der Studie teilnehmen können.

4.3 Direkt zu Beginn und am Ende der Studie wird bei jeder der 72 Studienteilnehmerinnen ein biofunktioneller Status (45 Tests) und eine Blut- und Urinentnahme durchgeführt. Dies dauert je eine Stunde. Der Inhalt des biofunktionellen Status wird im Kapitel 4.8 genauer beschrieben.

4.4 Die Teilnehmerinnen für die Studie werden nach der ersten Bestimmung des biofunktionellen Status gleichmässig in zwei Gruppen eingeteilt (Zufallsprinzip per Los), die Yogagruppe oder die Kontrollgruppe. Dabei können keine individuellen Wünsche berücksichtigt werden. Sie werden per Brief informiert, in welche Gruppe Sie eingeteilt wurden.

Yogagruppe:

Während der Studie werden die Teilnehmerinnen der Yogagruppe wöchentlich:

- An einer 90-minütigen online Iyengar-Yogalektion teilnehmen.
- Yogaübungen (2x 45 Minuten pro Woche) anhand von einer Foto- Übungssequenzen selbständig zu Hause durchführen und die eigene Übungspraxis in einem Tagebuch dokumentieren.

Weitere Informationen:

- Die Lektionen finden jeweils per Zoom-Sitzung in deutscher Sprache statt.
- Für den online Unterricht wird ein (eigenes) elektronisches Gerät mit Kamerafunktion (z. B. Laptop, iPad, Smartphone) benötigt.
- Das online-Yogaprogramm wird von der international bekannten Iyengar-Yoga-Lehrerin Rita Keller angeleitet (Co-Autorin des Buches Iyengar-Yoga in der Menopause) sowie von ihrer Vertretungslehrerin Gabi Göller.
- Iyengar-Yoga beinhaltet aktive Übungen (wobei das Level auf Ihre individuelle Beweglichkeit angepasst werden kann) Entspannungsübungen und Atemübungen. Die Haltungen der Übungssequenzen werden in der ersten Yogalektion vorgestellt und geübt.
- Sie benötigen für die Übungspraxis zuhause idealerweise eine rutschfeste Matte (Gymnastik- oder Yogamatte), sowie einen Stuhl, einen Yoga - Gurt und zwei Wolldecken als Hilfsmittel und ein Stück freie Wand. Es besteht die Möglichkeit eine Yogamatte und einen Yoga - Gurt auszuleihen.

Kontrollgruppe:

- Die Teilnehmerinnen der Kontrollgruppe werden keine Yogastunden oder -Übungen erhalten.
- Sie werden gebeten ihren normalen Alltag fortzusetzen und bei den zwei Untersuchungen (Biofunktioneller Test und Blutentnahme) teilzunehmen. Ihre Teilnahme ist ebenso wichtig wie die der Yogagruppe, da wir die Resultate am Ende vergleichen wollen um den Einfluss von Yoga darzustellen.

- Die Teilnehmerinnen der Kontrollgruppe erhalten nach Abschluss der Studie einen Gutschein für vier gratis Online-Yogakurse.

4.5 Abschlussgespräch

Am Ende der Studie werden Ihnen in einem ärztlichen Gespräch ihre individuellen Ergebnisse erklärt.

4.6 Zeitaufwand

Total erwartet Sie ein Zeitaufwand von mindestens 1 Stunde unabhängig ob Sie an der Studie teilnehmen oder nicht.
Bei den Studienteilnehmerinnen beträgt der Zeitaufwand mindestens 3 Stunden (Kontrollgruppe) und maximal 36 Stunden (Yoga-Gruppe) über 3-4 Monate während der Zeit vom Screening bis zum Ende der Studie.

4.7 Die Voruntersuchung beinhaltet:
- Gespräch: Rauchen, Herzerkrankungen, Hirnschlag, Diabetes, Bluthochdruck, Alkoholkonsum
- Untersuchung: Blutdruck, Puls, Temperatur, BMI
- Blutentnahme: Schilddrüsenhormone, Blutfette, Entzündungseiweiss, Stresslevel, Hormone
- Fragebögen: Beschwerden nach der Menopause, Umfrage zu Angst und Depression

4.8 Der biofunktionelle Status beinhaltet:
- Physikalisches: Blutdruck, Puls (Ruhe und unter Belastung), Lungenfunktion, Handkraft, Messung der Zellmasse, Anzahl Zähne
- Sensorik und Psychomotorik: Sehschärfe, Hörtest, Motivation, Ausdauer
- Kognitives und Mentales: Verschiedene Reaktionszeiten, Konzentration, strategisches Denken, Gedächtnis, Orientierung
- Emotionales und Soziales: Fragebogen, Stress im Alltag, Selbstkontrolle

4.9 Die Blutentnahme beinhaltet:
- Entzündungsmarker und Blutfettwerte, die als Risikofaktoren für eine Erkrankung der Herzkranzgefässe gelten.
- Sexualhormone (Östrogen und Progesteron)

Die Urinprobe beinhaltet:
- Marker die als Risikofaktoren für eine Erkrankung der Herzkranzgefässe gelten.

Zusammenfassung:

**Besuch**	Information	Screening	1. Besuch	Yogaintervention oder Kontrolle	2. Besuch
**Mündliche und schriftliche Patienteninformation**	+				
**Schriftliche Einwilligung**		+			
**Einschluss- und Ausschluss-Evaluation**		+			
**Gespräch**		+			
**Körperliche Untersuchung**		+			
**Blutentnahme**		+			
**Biofunktioneller Status**			+		+
**Yoga oder Kontrolle**				+	
**Fragebogen**		+	+		+
**Blutentnahme (HS-CRP, Hormone)**			+		+
**Urinprobe**			+		

5. Ausschluss

Es kann sein, dass wir Sie von der Studie vorzeitig ausschliessen müssen. Das kann geschehen, wenn Sie unsere Einschlusskriterien nicht- oder mindestens eines der Ausschlusskriterien erfüllen (siehe Punkt 2).

6. Nutzen

Durch die Studie erhalten die Teilnehmerinnen die Gelegenheit mehr über sich selbst herauszufinden. Durch den biofunktionellen Status und das kostenlose Beratungsgespräch am Ende erhalten Sie Einsicht in Ihre physikalischen, psychologischen, psychomotorischen, emotionalen, sozialen, kognitiven und mentalen Fähigkeiten. Durch den Vergleich Ihrer Resultate mit der gleichaltrigen mitteleuropäischen Bevölkerung können Sie sehen, wo Ihre Fähigkeiten stark ausgebildet sind (Stärken) und an welchen Sie noch arbeiten können (Ressourcen). Teilnehmerinnen des Yogaprogramms erhalten 3 Monate lang kostenlosen, hoch qualifizierten Iyengar-Yogaunterricht durch ausgebildete Yogalehrerinnen. Die Teilnehmerinnen der Kontrollgruppe erhalten die Möglichkeit nach Abschluss der Studie 4x gratis an einem Online-Yogakurs teilzunehmen.

7. Rechte

Sie nehmen freiwillig teil. Wenn Sie nicht mitmachen oder später Ihre Teilnahme zurückziehen wollen, müssen Sie dies nicht begründen. Ihre medizinische Behandlung/Betreuung ist unabhängig von Ihrem Entscheid gewährleistet. Sie dürfen jederzeit Fragen zur Studienteilnahme stellen. Wenden Sie sich dazu bitte an die Person, die am Ende dieser Information genannt ist.

8. Pflichten

Als Teilnehmer ist es notwendig, dass Sie

- sich an die notwendigen Vorgaben und Anforderungen der Studie durch den Prüfplan halten. Dies heisst, Sie:
- Erscheinen an allen individuell vereinbarten Untersuchungsterminen (Screening, biofunktionelle Status und Blut- und Urinentnahme)
- Nehmen an mindestens 10 von 12 Yogalektionen teil (falls in Yoga-Gruppe).

- Führen selbständig die Yoga-Übungen zu Hause durch (falls in Yoga-Gruppe) und dokumentieren dies im Tagebuch.

- Bitte informieren Sie Ihren Prüfarzt über den Verlauf Ihres Befindens und melden zeitnah neue Symptome, oder neue Beschwerden.
- Sie werden ausserdem gebeten, Ihren Prüfarzt über die gleichzeitige Behandlung und Therapie bei einem anderen Arzt und über die Einnahme von Medikamenten zu informieren.

9. Risiken und Belastungen für die Teilnehmenden

Die Teilnahme am Yogaprogramm sowie der biofunktionelle Status beinhaltet keine relevanten Risiken.

10. Andere Behandlungsmöglichkeiten

Sie müssen bei dieser Studie nicht teilnehmen. Wenn Sie nicht teilnehmen möchten, können Sie sich bei (post-)menopausalen Beschwerden an Ihre/n Gynäkologin/e wenden. Dieser Berät Sie gerne bezüglich anderweitigen Behandlungsmöglichkeiten.

11. Ergebnisse aus der Studie

Der Prüfarzt wird Sie während der Studie über alle neuen Erkenntnisse informieren, die den Nutzen der Studie oder Ihre Sicherheit und somit Ihre Einwilligung zur Teilnahme an der Studie beeinflussen können. Sie werden die Information mündlich und schriftlich erhalten.
Bei Zufallsbefunden (Screening oder sonstigen Blut- oder Urinentnahmen) die bei Ihnen zur Verhinderung, Feststellung und Behandlung bestehender oder künftig zu erwartenden Krankheiten beitragen können, werden Sie informiert. Wenn Sie nicht informiert werden wollen (sog. Recht auf Nicht-Wissen), sprechen Sie bitte mit Ihrem Prüfarzt.

12. Vertraulichkeit der Daten und Proben

Für diese Studie werden Ihre persönlichen und medizinischen Daten erfasst. Nur sehr wenige Fachpersonen werden Ihre unverschlüsselten Daten sehen, und zwar ausschliesslich, um Aufgaben im Rahmen der Studie zu erfüllen. Bei der Datenerhebung zu Studienzwecken werden die Daten verschlüsselt. Verschlüsselung bedeutet, dass alle Bezugsdaten, die Sie persönlich identifizieren könnten (Name, Geburtsdatum), gelöscht und durch einen Schlüssel ersetzt werden. Die Schlüssel-Liste bleibt immer im Spital. Diejenigen Personen, die den Schlüssel nicht kennen, können daher keine Rückschlüsse auf Ihre Person ziehen. Bei einer Publikation sind die zusammengefassten Daten daher auch nicht auf Sie als Einzelperson zurückverfolgbar. Ihr Name taucht niemals im Internet oder einer Publikation auf. Manchmal gibt es die Vorgabe bei einer Zeitschrift zur Publikation, dass Einzel-Daten (sogenannte Roh-Daten) übermittelt werden müssen. Wenn Einzel-Daten übermittelt werden müssen, dann sind die Daten immer verschlüsselt und somit ebenfalls nicht zu Ihnen als Person zurückverfolgbar. Die Vorgaben des Datenschutzes werden eingehalten und Sie als teilnehmende Person haben jederzeit das Recht auf Einsicht in Ihre Daten.
Wenn Daten/ Proben vor Ort gelagert werden, handelt es sich um eine Datenbank/ Biobank für Forschungszwecke.

Die Auswertung der verschlüsselten Daten findet durch den Statistiker, Dr. rer. nat. Normann Bitterlich in Chemitz, Deutschland statt.

.
Möglicherweise wird diese Studie durch die zuständige Ethikkommission oder durch die Institution, die die Studie veranlasst hat, überprüft. Der Prüfarzt muss eventuell Ihre persönlichen und medizinischen Daten für solche Kontrollen offenlegen.

13. Rücktritt

Sie können jederzeit von der Studie zurücktreten, wenn Sie das wünschen. Die bis dahin erhobenen Daten und Proben werden verschlüsselt und ausgewertet. Nach der Auswertung werden sie anonymisiert.

14. Entschädigung für Teilnehmende
Sie erhalten keine finanzielle Entschädigung für die Teilnahme an der Studie. Die Teilnahme an dieser Studie ist für Sie jedoch kostenlos.

15. Urheberrecht
Die Aufzeichnungen und Inhalte des Yogaunterrichts sind geistiges Eigentum der Yogalehrerinnen. Jede Weitergabe an Dritte ist untersagt.

16. Haftung
Die Universitätsklinik für Frauenheilkunde des Inselspitals haftet für Schäden, welche Ihnen im Zusammenhang mit der getesteten Substanz oder Forschungshandlungen (z.B. Untersuchungen) entstehen könnten. Die Voraussetzungen und das Vorgehen dazu sind gesetzlich geregelt.
Falls Sie einen Schaden erlitten haben, so wenden Sie sich bitte an den Prüfarzt.

17. Finanzierung der Studie
Die Studie wird von der Schweizerischen Iyengar-Yogavereinigung finanziell unterstützt. Ein Teil der Studie wird vom Sponsor/ Investigator selbst bezahlt.

18. Kontaktperson(en)
Bei Fragen, Unsicherheiten oder Notfällen, die während der Studie oder danach auftreten, können Sie sich jederzeit an eine dieser Kontaktpersonen wenden.

Prof. Dr. med. Petra Stute
Leitende Ärztin und Stv. Leiterin der Abtl. für Gynäkologische Endokrinologie und Reproduktionsmedizin
Universitätsklinik für Frauenheilkunde Inselspital Bern
Friedbühlstrasse 19
CH - 3010 Bern
Tel.: +41 31 632 13 03
E-mail: petra.stute@insel.ch

Sofie Schelker
sofie.schelker@students.unibe.ch
+41 79 761 21 02

19. Glossar (erklärungsbedürftige Begriffe);

- Was heisst „randomisiert“?
  Bei vielen Studien werden zwei oder mehrere unterschiedliche Arten der Behandlung verglichen. „Randomisieren“ bedeutet dann, dass ausgelost wird, wer in welche Gruppe kommt. Es ist bei einem solchen Test also Zufall, ob man das echte Medikament erhält oder das Placebo.

- Was heisst "einfachblind"?
  Eine Studie zu "verblinden" (einfach) dient dazu, bessere und genauerer Ergebnisse zu erhalten. Von einer "einfachblinden" Studie spricht man, wenn bei der Studie entweder die Studienteilnehmer oder die Forschenden nicht wissen, ob ein Teilnehmer das echte oder das Scheinmedikament erhält. Wer was bekommt, lost eine Person aus, die nicht bei dem Test mitmacht.
  In unserer Studie werden die Forscher verblindet werden, so können sie die Resultate nicht in eine Richtung beeinflussen und behandeln alle Teilnehmerinnen gleich.
  Wenn der Test zu Ende ist, wird die "Verblindung" aufgelöst. In einem Notfall kann die "Verblindung" jederzeit auch früher aufgehoben werden.

Einwilligungserklärung

Schriftliche Einwilligungserklärung zur Teilnahme an einem Studienprojekt
Bitte lesen Sie dieses Formular sorgfältig durch. Bitte fragen Sie, wenn Sie etwas nicht verstehen oder wissen möchten. Für die Teilnahme ist Ihre schriftliche Einwilligung notwendig.

**BASEC-Nummer (nach Einreichung):**	
**Titel der Studie**<br>**(wissenschaftlich und Laiensprache)**:	Influence of Iyengar yoga on the bio-functional age of postmenopausal women<br><br>Einfluss von Iyengar-Yoga auf das biofunktionelle Alter von Frauen nach der Menopause
**verantwortliche Institution**<br>**(Sponsor mit Adresse)**:	Universitätsklinik für Frauenheilkunde Inselspital Bern<br>Friedbühlstrasse 19<br>CH - 3010 Bern
**Ort der Durchführung**:	Universität für Frauenheilkunde, Inselspital Bern
**Verantwortlicher Prüfarzt am Studienort:**<br>Name und Vorname in Druckbuchstaben:	Prof. Dr. med. Petra Stute
**Teilnehmerin**<br>Name und Vorname in Druckbuchstaben:<br>Geburtsdatum:	☐ weiblich

- Ich wurde vom unterzeichnenden Prüfarzt/Prüfärztin mündlich und schriftlich über den Zweck, den Ablauf der Studie mit der Yoga-Intervention und über mögliche Vor- und Nachteile sowie über eventuelle Risiken informiert.
- Ich nehme an dieser Studie freiwillig teil und akzeptiere den Inhalt der abgegebenen schriftlichen Information. Ich hatte genügend Zeit, meine Entscheidung zu treffen.
- Meine Fragen im Zusammenhang mit der Teilnahme an dieser Studie sind mir beantwortet worden. Ich behalte die schriftliche Information und erhalte eine Kopie meiner schriftlichen Einwilligungserklärung.
- Ich bin einverstanden, dass die zuständigen Fachleute des Sponsors, der zuständigen Ethikkommission und zu Prüf- und Kontrollzwecken in meine unverschlüsselten Daten Einsicht nehmen dürfen, jedoch unter strikter Einhaltung der Vertraulichkeit.
- Bei Studienergebnissen oder Zufallsbefunden, die direkt meine Gesundheit betreffen, werde ich informiert. Wenn ich das nicht wünsche, informiere ich meinen Prüfarzt.
- Ich weiss, dass meine gesundheitsbezogenen und persönlichen Daten (und Proben) nur in verschlüsselter Form zu Forschungszwecken für diese Studie weitergegeben werden können
- Ich kann jederzeit und ohne Angabe von Gründen von der Studienteilnahme zurücktreten. Meine weitere medizinische Behandlung ist unabhängig von der Studienteilnahme immer gewährleistet. Die bis zum Rücktritt erhobenen Daten und Proben werden für die Auswertung zur Studie verwendet.
- Die Haftpflichtversicherung des Spitals/der Institution kommt für allfällige Schäden auf.

- Ich bin mir bewusst, dass die in der Teilnehmerinformation genannten Pflichten einzuhalten sind. Im Interesse meiner Gesundheit kann mich der Prüfarzt jederzeit von der Studie ausschliessen.

Ort, Datum	Unterschrift Teilnehmerin

Bestätigung des Prüfarztes/der Prüfperson: Hiermit bestätige ich, dass ich dieser Teilnehmerin/ diesem Teilnehmer Wesen, Bedeutung und Tragweite der Studie erläutert habe. Ich versichere, alle im Zusammenhang mit dieser Studie stehenden Verpflichtungen gemäss dem geltenden Recht zu erfüllen. Sollte ich zu irgendeinem Zeitpunkt während der Durchführung der Studie von Aspekten erfahren, welche die Bereitschaft der Teilnehmerin zur Teilnahme an der Studie beeinflussen könnten, werde ich sie/ ihn umgehend darüber informieren.

Ort, Datum	Name und Vorname der Prüfärztin/ des Prüfarztes in Druckbuchstaben<br><br>Unterschrift der Prüfärztin/des Prüfarztes
