# Supplementary material for: G.A study protocol for a randomized controlled trial investigating the influence of Iyengar Yoga on biofunctional age and cardiovascular risk associated biomarker of postmenopausal women
Source: Front Glob Womens Health. 2026 Mar 16;7:1762048. doi: 10.3389/fgwh.2026.1762048 (PMC13033725; doi:10.3389/fgwh.2026.1762048)
Supplement: Supplementary File S4 — Program of the Yoga-Sessions made at home (.pdf) [file Datasheet4.pdf]

INSELSPITAL
UNIVERSITÄTSSPITAL BERN
HÔPITAL UNIVERSITAIRE DE BERNE

Exercise program for participants in the yoga program as part of the Inselspital Bern study

Influence of Hatha Yoga on the biofunctional age and cardiovascular risk of postmenopausal women

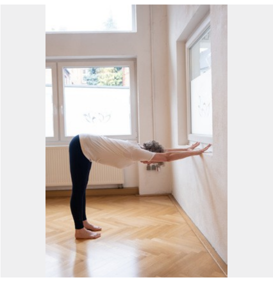

Ardha Uttanasana

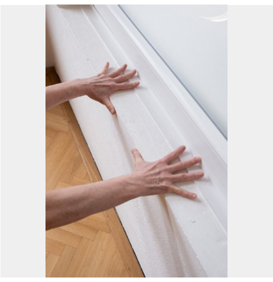

Hands in Ardha Uttanasana

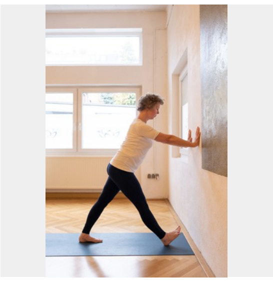

Parshvottanasana

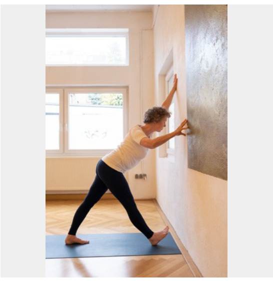

Left arm raised, hand on wall

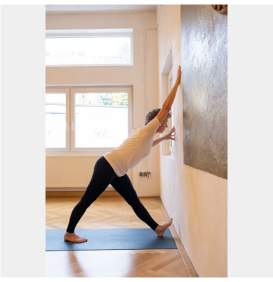

Right arm raised, hand on wall

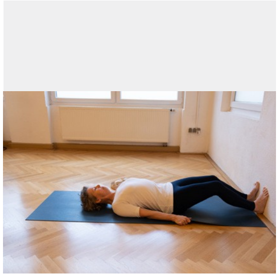

Supta Tadasana, feet on wall, legs bent, stretch with resistance to:

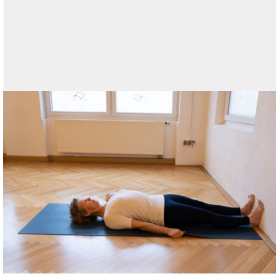

Supta Tadasana, feet on wall

All photos copyright © Rita Keller, Light on Yoga Academy
www.light-on-yoga.org | info@light-on-yoga.org

INSELSPITAL
UNIVERSITÄTSSPITAL BERN
HÔPITAL UNIVERSITAIRE DE BERNE

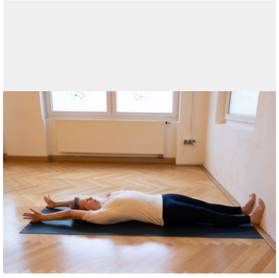

Urdhva Hastasana in Supta Tadasana, palms facing each other

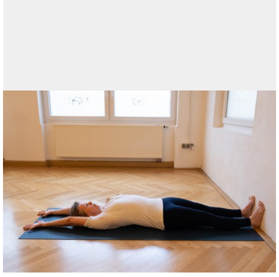

Urdhva Hastasana in Supta Tadasana, backs of hands on the floor

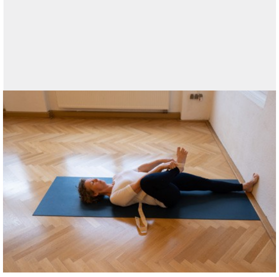

Supta Padangushthasana 1, on the right side

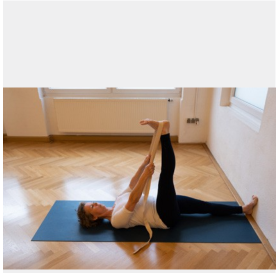

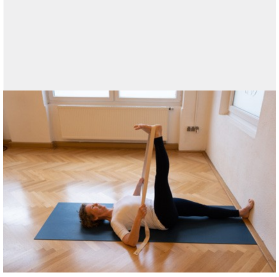

Supta Padangushthasana 1, arms bent, chest expanded

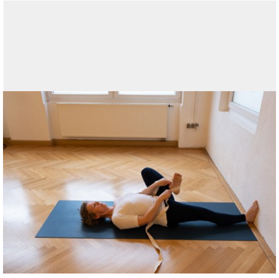

Supta Padangushthasana 1, on the left side

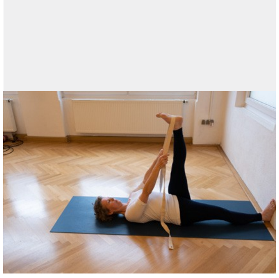

All photos copyright © Rita Keller, Light on Yoga Academy
www.light-on-yoga.org | info@light-on-yoga.org

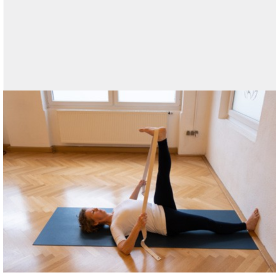

Supta Padangushthasana, arms bent, chest expanded

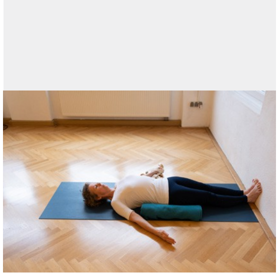

Supta Padangushthasana 2 on the right side

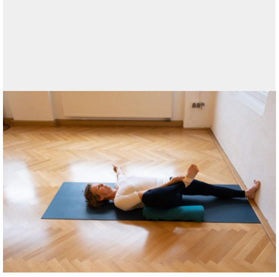

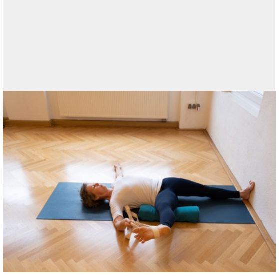

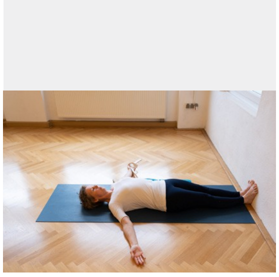

Supta Padangushthasana 2 on the left side

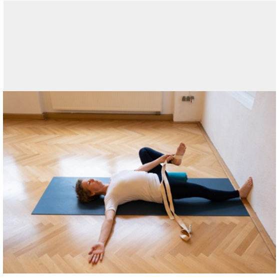

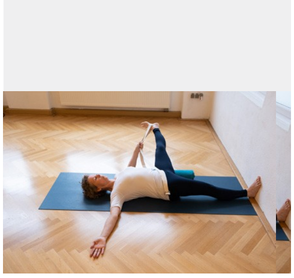

All photos copyright © Rita Keller, Light on Yoga Academy
www.light-on-yoga.org | info@light-on-yoga.org

INSELSPITAL
UNIVERSITÄTSSPITAL BERN
HÔPITAL UNIVERSITAIRE DE BERNE

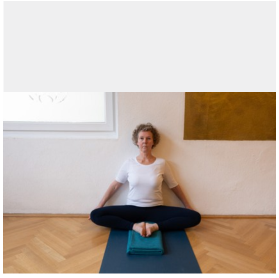

Baddha Konasana with wall

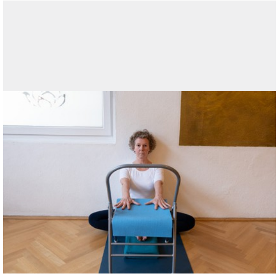

Baddha Konasana with wall + chair, hands on seat

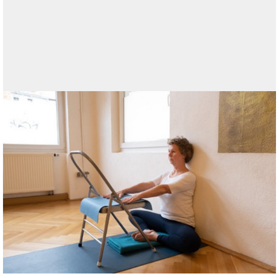

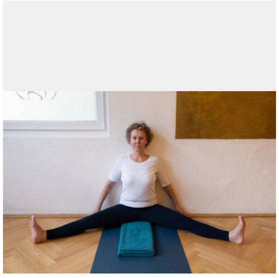

Upavishtha Konasana with wall

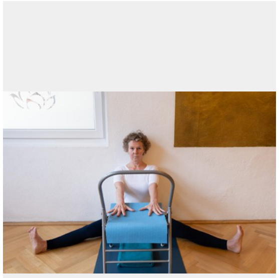

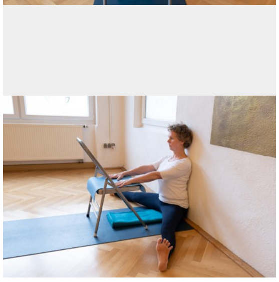

All photos copyright © Rita Keller, Light on Yoga Academy
www.light-on-yoga.org | info@light-on-yoga.org

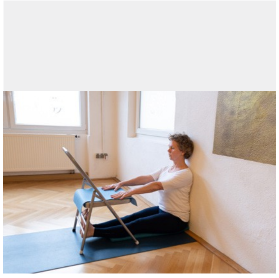

Dandasana

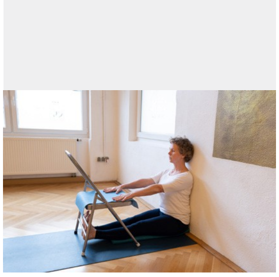

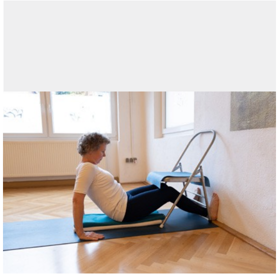

Setu Bandha Sarvangasana, setup - 2 blankets, feet against wall

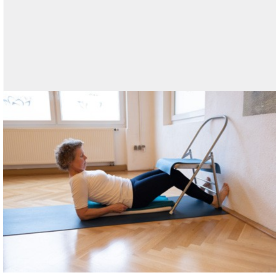

Setu Bandha Sarvangasana, legs bent, hands grasping the mat at the sides

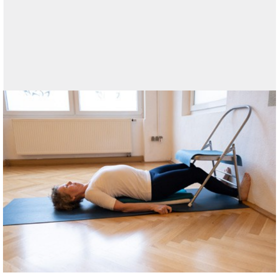

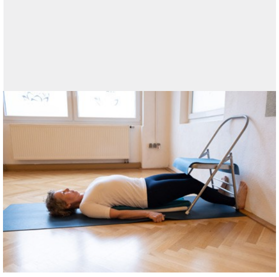

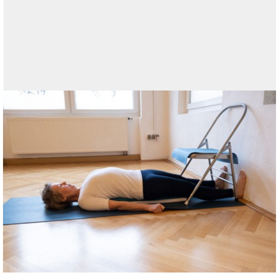

All photos copyright © Rita Keller, Light on Yoga Academy
www.light-on-yoga.org | info@light-on-yoga.org

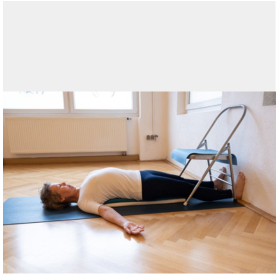

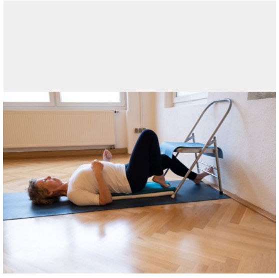

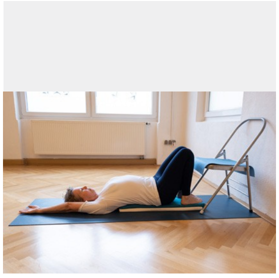

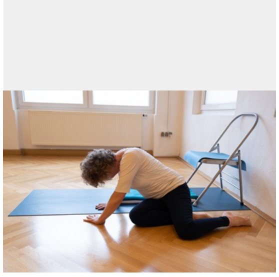

Come up via the side

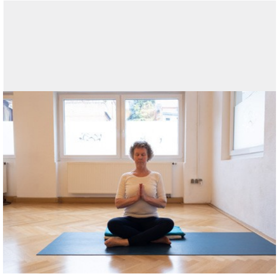

Sit upright, hands in namaste

All photos copyright © Rita Keller, Light on Yoga Academy
www.light-on-yoga.org | info@light-on-yoga.org

What do I need to bear in mind when practicing at home?

For our study, it is important that, in addition to participating online, you also do yoga exercises at home twice a week for about 45 minutes.

- Please document your own practice in the journal we have provided for you.
- You can use this photo sequence or the recordings of the online class for your own practice. The program at home should consist of approximately 30 minutes of active postures and approximately 15 minutes of relaxation.
- When practicing at home, please stick to yoga exercises that you were able to do well online and that made you feel comfortable. You are welcome to omit any yoga poses that you found too difficult.

See also "Information for participants in the yoga program."
