# Supplementary material for: G.A study protocol for a randomized controlled trial investigating the influence of Iyengar Yoga on biofunctional age and cardiovascular risk associated biomarker of postmenopausal women
Source: Front Glob Womens Health. 2026 Mar 16;7:1762048. doi: 10.3389/fgwh.2026.1762048 (PMC13033725; doi:10.3389/fgwh.2026.1762048)
Supplement: Supplementary File S5 — Biofunctional Status (.pdf) [file Datasheet5.pdf]

Supplementary file 4: Biofunctional Status

Physical strengths and resources	*Value*	*Test*
- Systolic and diastolic blood pressure	mmHg	Sphyngometry
- Resting heart rate	1/min	Submaximal Ergometry (Squad)
- Pulse rate difference	1/min	Submaximal Ergometry (Squad)
- Performance pulse index		Submaximal Ergometry (Squad)
- Performance time	sec	Submaximal Ergometry (Squad)
- Vital capacity (FVC)	%	Spirometry (custo spiro mobile)
- Hand grip strength (both sides)	KP	Dynamometry
- Fat mass	kg	Bioimpedance analysis (BIA)
- Body cell mass	kg	Bioimpedance analysis (BIA)
- Decayed missing filled teeth	Pieces	Teeth status

Sensory physiological and psychomotor strength		
- Vision right and left	%	Vision testing (5m distance)
- Hearing loss right and left 2048 Hz	%	Audiometry (2000 and 4000 Hz)
- Hearing loss right and left 4096 Hz	%	Audiometry (2000 and 4000 Hz)
- Psychomotor activity: start and basic rate		Tapping (0 sec, 10 sec, 60 sec)
- Test motivation		Tapping (0 sec, 10 sec, 60 sec)
- Psychomotor endurance		Tapping (0 sec, 10 sec, 60 sec)
- Viseomotor coordination ability (time)	sec	Tracking
- Viseomotor coordinance ability (mistakes)		

Cognitive and mental strength and resources		
- Optical reaction time	msec	Optical response (10 times)
- Acoustical reaction time	msec	Acoustical response (10 times)
- Pursuing reaction time	msec	Pursuing reaction time (10 times)
- Verbal reaction time	sec	Color-word-test (Stroop)
- Cognitive reaction time	sec	Color-word-test (Stroop)
- Cognitive switch capability	sec	Color-word-test (Stroop)
- Ability to concentrate (Time and mistakes)	sec	Concentration-time-test (Landolt)
- Strategic thinking	sec	Stepping stome maze
- Memory performance		Stepping stome maze
- Orientation capacity		Stepping stome maze
- Change over capability		Stepping stome maze
Emotional-social strength and resources		
- Physical wellbeing		Health complaint questionnaire (Hoeck/ Hess)
- Emotional wellbeing		Health complaint questionnaire (Hoeck/ Hess)
- Sens of coherence		SOCL9
- Stress exposition/ social resonance		Giessen-Test
- Social dominance		Giessen-Test
- Social power		Giessen-Test
- Stress disposition/ self-control		Giessen-Test
- Social activity (duties, leisure)		Social activity questionnaire
